# Effectiveness of educational intervention on breast cancer knowledge and breast self-examination among female university students in Bangladesh: a pre-post quasi-experimental study

**DOI:** 10.1186/s12885-022-09311-y

**Published:** 2022-02-22

**Authors:** Rumpa Sarker, Md. Saiful Islam, Mst. Sabrina Moonajilin, Mahmudur Rahman, Hailay Abrha Gesesew, Paul R. Ward

**Affiliations:** 1 Department of Public Health and Informatics, Jahangirnagar University, Savar, Dhaka, Bangladesh; 2 Centre for Advanced Research Excellence in Public Health, Savar, Dhaka, Bangladesh; 3 Department of Epidemiology, School of Health Sciences, Mekelle University, Mekelle, Ethiopia; 4 Centre for Health Policy Research, Torrens University, Adelaide, SA Australia

**Keywords:** Breast cancer, Breast self-examination, Educational intervention, Female, University

## Abstract

**Background:**

Breast cancer is a global health issue and a leading cause of death among women. Early detection through increased awareness and knowledge on breast cancer and breast cancer screening is thus crucial. The aim of the present study was to assess the effect of an educational intervention program on breast cancer knowledge and the practice of breast self-examination among young female students of a university in Bangladesh.

**Methods:**

A quasi-experimental (pre-post) study design was conducted at Jahangirnagar University in Bangladesh. Educational information on breast cancer and breast self-examination (BSE), demonstration of BSE procedure and leaflets were distributed among 400 female students after obtaining written informed consent. The stepwise procedures of BSE performance were demonstrated with images. Pre-intervention and 15 days post-intervention assessments were conducted to assess the changes in knowledge on breast cancer and practices of BSE. Mc-Nemar’s tests and paired sampled *t*-tests were performed to investigate the differences between pre- and post-test stages.

**Results:**

A total of 400 female university students aged 18-26 years were included in the sample. Significant changes were found in knowledge and awareness about breast cancer and BSE practices after the educational intervention. The significant differences were measured in the mean scores of pre-test vs. post-test: breast cancer symptoms (2.99 ± 1.05 vs. 6.35 ± 1.15; *p* < 0.001), risk factors (3.35 ± 1.19 vs. 7.56 ± 1.04; *p* < 0.001), treatment (1.79 ± 0.90 vs. 4.63 ± 0.84; *p* < 0.001), prevention (3.82 ± 1.32 vs. 7.14 ± 1.03; *p* < 0.001), screening of breast cancer (1.82 ± 0.55 vs. 3.98 ± 0.71; *p* < 0.001) and process of BSE (1.57 ± 1.86 vs. 3.94 ± 0.93; *p* < 0.001). Likewise, a significant percentage of change in BSE practices was obtained between pre-test and post-test (21.3% vs. 33.8%; *p* < 0.001).

**Conclusions:**

Study findings confirm that the study population had inadequate awareness and knowledge at baseline which was improved significantly after educational intervention. A nationwide roll-out with community-based interventions is recommended for the female population in both rural and urban areas.

**Supplementary Information:**

The online version contains supplementary material available at 10.1186/s12885-022-09311-y.

## Introduction

Breast cancer is a worldwide health concern and one of the most prominent causes of mortality among women. In 2018, approximately 2 million new breast cancer cases were detected, which is approximately 23% of all cancers, the most occurring cancer among women [1]. In Bangladesh, breast cancer is ranked as the second most leading cancer after cervical carcinoma and in females, these two cancers constitute 38% of all cancers [2]. This continuously rising burden has been a matter of concern for a long time, especially for limited resourced countries like Bangladesh [3]. As curative treatment for any cancer is yet not available, several approaches have been advocated towards increasing awareness that may lead to early detection of cancers including breast cancer [4]. Breast cancer education and awareness in limited resourced countries can be a key to initiate the early detection of breast cancer and subsequently increase the survival rate [5]. Numerous findings suggest that educating the community about assessment of asymptomatic women has the potential to increase the proportion of breast cancer detected at an early stage. Studies conducted with female students in Turkey, Malaysia and India have showed significantly improved knowledge and awareness of breast cancer after educational interventions using various health educational tools such as group discussion sessions, video demonstration and pamphlets [6–8]. Findings from a pilot mobile intervention program in Bangladesh has reported that, in comparison with a control group, the women who attended to an educational intervention were more likely to visit clinics for a follow-up to check for abnormalities found in their breast examination [9]. This strengthens the vital role of education in decreasing late presentation of breast cancer.

Recommended screening methods like mammograms, clinical breast examination, ultrasounds and MRIs are not financially feasible to implement as a nationwide screening program in low-resource countries like Bangladesh. Moreover, lack of knowledge and awareness about breast cancer has been reported from some studies conducted with females in Bangladesh which may contribute to less adherence of women to receive recommended screening [10–13]. Therefore, improving breast cancer awareness and breast self-examination (BSE) through educational interventions among females may be a feasible solution to early detection. However, in order to assess the impact of an intervention, we need to know the present level of knowledge, attitude and practices of the female population towards breast cancer and BSE. Unfortunately, in Bangladesh, the currently available data is limited. Consequently, this study was planned to assess the knowledge and practice level of breast cancer and BSE among female university students (pre-test) and to note the changes in knowledge of risk factors, symptoms, diagnosis and treatment modalities of breast cancer, and to know about practice of BSE (pre-test vs. post-test) in females after an educational intervention. The young female university students aged from 18 to 26 are already passing their reproductive age and are the future mothers. Also, they are considered to be the most educated segment of the population. Firstly, it may create positive impact and increase the awareness about breast cancer. Secondly, as they belong to the most educated population, they can help in spreading the knowledge and awareness among their own family, friends and community in large.

## Methodology

### Study design and setting

A pre-post quasi-experimental interventional study was conducted among female university students residing in dormitories of Jahanginagar University in Dhaka, Bangladesh from December 2019 to March 2020. Jahanginagar University is the largest and only fully residential university in Bangladesh.

### Participants and procedures

The study was conducted among 400 female respondents of aged 18-26 years corresponding to Honours 1st year to master’s students. Inclusion criteria included: being female students residing in the university’s dormitories and being aged 18-26 years old. Exclusion criterion was being female students who didn’t reside within residential dormitories. Initially, Yamane’s simplified sampling formula was employed to determine a sample size and a total of 386 participants were estimated. However, we gathered 400 responses in order to make sure that our final sample size was large enough to detect statistical differences pre-post intervention. The proportionate stratified random sampling technique was conducted to calculate the study sample from each dormitory. In this approach, each stratum sample size was directly proportional to the population size of the entire population of the strata. The study was carried out in three phases: first phase (pre-intervention phase), second phase (intervention phase) and third phase (post-intervention phase).

#### First phase (pre-intervention phase)

A pre-designed structured interview questionnaire was used to collect the following data from the respondents: socio-demographic data, respondent’s knowledge, attitude and practice regarding breast cancer, screening and BSE.

#### Second phase (intervention phase)

Materials (e.g. lecture/discussions, brain storming, leaflets showing images of the stepwise process of BSE, etc.) were used during the interventional phase. All the sessions were conducted in the respective dormitories of the respondents. Participants were divided into groups of 10-15 people to conduct the sessions so that the educational intervention could be clearly delivered to and understood by the participants. Each session took 45-60 min. After the pre-test session, the participants were given a short break to rearrange themselves into divided groups and get prepared for the educational session. Both pre-test and intervention sessions were conducted on the same day. Each participant was assigned a unique ID number so that they could be traced back for the post-test session. To ensure that the respondents could understand the educational materials, in every session one or two respondents from each group were encouraged to demonstrate and share what they had learned. This was also chosen randomly among the participants who were willing to perform this task.

#### Third phase (post-test phase)

Fifteen days after the education session, participants were re-contacted for a post-test survey. During post-test phase, participants were exposed to the same questions in the pre-test questionnaire to assess any changes in knowledge about breast cancer and practices of BSE.

### Study instruments

A pre-tested and semi-structured questionnaire including informed consent, socio-demographic information and questions related to knowledge towards breast cancer and BSE practices, was prepared for the study through extensive literature review [14–17].

The questionnaire was reviewed by an external reviewer who was an oncologist and had sound knowledge about breast cancer. Likewise, a pilot test was conducted with 20 participants to assess the readability of the questionnaire. The questionnaire was finalized after incorporating minor amendments based on participant feedback during the pilot study. A paper-pen-based survey was conducted among participants. Additionally, a few post-test surveys were undertaken via telephone from respondents who could not be present during the post-test session.

#### Socio-demographic information

Socio-demographic information was recorded during the survey including age, study year (1st/2nd/3rd/4th/Master’s), marital status (unmarried/married), family history of breast cancer (yes/no), and relationship with breast cancer affected patient (mother/sister/cousin/aunt/grandmother).

#### Knowledge of breast cancer

To assess participant knowledge of breast cancer, a total of 43 questions (i.e., 8 for symptoms, 10 for risk factors, 6 for treatment, 8 for prevention, 5 for screening, and 5 for process of BSE) were asked during the survey. All questions were answered with three possible responses (i.e., yes/no/don’t know). During analysis, ‘yes’ responses were coded as ‘1’; while ‘no’ or ‘don’t know’ responses were scored as ‘0’. To get the total score of a construct (e.g., symptoms, risk factors, treatment, prevention, screening, and process of BSE), the raw scores from each question were summated. The greater scores indicate the more knowledge. The distributions of all questions (both pre-test and post-test) are presented in Additional file.

#### BSE practices

A single question “*Have you ever self-examined your breast for breast cancer?”* was used to assess the BSE with binary responses (yes/no).

### Statistical analysis

The SPSS version 25.0 was used for processing and analyzing data. Descriptive statistics were performed. To assess the differences between pre-test and post-test, Mc-Nemar tests and paired sample t-tests were computed as appropriate. Before performing the Mc-Nemar tests, each question was transformed into dichotomous (i.e., correct answer and wrong answer). A *p*-value less than 0.05 was deemed as statistically significant.

### Ethical consideration

The study was conducted in accordance with the Institutional Research Ethics guidelines and ethical principle involving human participation (i.e., Helsinki Declaration). Formal ethics approval was granted by the Biosafety, Biosecurity, and Ethical Clearance Committee, Jahangirnagar University, Savar, Dhaka-1342, Bangladesh. At first, all participants were informed about the purpose and objectives of the study. Participants were informed that it was a three-phase study, and also about the duration of the study and the approximate time that would be taken from them. Then, written informed consents were taken from each of them who agreed to participate in the study. All information related to participants was kept confidential.

## Results

### General characteristics of participants

The sample comprised of a total of 400 female university students aged 18-26 years (see Table 1 for details of the participants). In terms of a family history of breast cancer, 18.3% participants reported that someone in their family had been diagnosed with the disease which included mother (11.6%), sister/cousin (24.6%), aunt (40.6%) and grandmother (23.2%). The remaining 81.2% had no family history of breast cancer.

**Table 1:** Socio-demographic variables

Variables	n	(%)
**Age**		
18-20	141	(35.3)
21-23	124	(31.0)
24-26	135	(33.8)
**Study year**		
1st	78	(19.5)
2nd	94	(23.5)
3rd	87	(21.8)
4th	84	(21.0)
Master’s	57	(14.2)
**Marital status**		
Unmarried	344	(86.0)
Married	56	(14.0)
**Family history of breast cancer**		
Yes	73	(18.3)
No	327	(81.8)
**Relationship with affected patient**		
Mother	8	(11.6)
Sister/cousin	17	(24.6)
Aunt	28	(40.6)
Grandmother	16	(23.2)

### Effectiveness of intervention on breast cancer knowledge and BSE

The participants could be traced back successfully and 100% response rate was obtained. Table 2 presents the overall differences in the participants’ knowledge regarding symptoms, risk factors, treatment, prevention, screening methods and process of BSE examination. Significant knowledge differences in the mean scores were obtained between pre-test and post-test: breast cancer symptoms (2.99 ± 1.05 vs. 6.35 ± 1.15; *p* < 0.001), risk factors (3.35 ± 1.19 vs. 7.56 ± 1.04; *p* < 0.001), treatment (1.79 ± 0.90 vs. 4.63 ± 0.84; *p* < 0.001), prevention (3.82 ± 1.32 vs. 7.14 ± 1.03; *p* < 0.001), screening of breast cancer (1.82 ± 0.55 vs. 3.98 ± 0.71; *p* < 0.001) and process of breast self-examination (1.57 ± 1.86 vs. 3.94 ± 0.93; *p* < 0.001). Likewise, a significate percentage of change in BSE practices was obtained between pre-test and post-test (21.3% vs. 33.8%; *p* < 0.001) (Table 3). The distribution and changes of the participants’ knowledge regarding symptoms, risk factors, treatment, prevention, screening methods and process of BSE examination are presented in Additional file (Tables S1-S5).

**Table 2:** Assessment of total difference in the knowledge of breast cancer among participants (pre-test vs. post-test)

Variables	Correct percentages	Mean	SD	(Min-Max)	***t***^**a**^	***P***-value
**Knowledge about symptoms of breast cancer (total score = 8)**						
Post-test	79.4%	6.35	1.15	(2.88–3.09)	42.343	< 0.001
Pre-test	37.4%	2.99	1.05	(6.24–6.46)		
Differences	42.0%	3.36	1.59	–		
**Knowledge about risks of breast cancer (total score = 10)**						
Post-test	75.6%	7.56	1.04	(3.23–3.47)	52.161	< 0.001
Pre-test	33.5%	3.35	1.19	(7.46–7.67)		
Differences	42.1%	4.21	1.62	–		
**Knowledge about treatment of breast cancer (total score = 6)**						
Post-test	77.2%	4.63	0.84	(1.70–1.88)	45.211	< 0.001
Pre-test	29.8%	1.79	0.90	(4.54–4.71)		
Differences	47.3%	2.84	1.26	–		
**Knowledge about prevention of breast cancer (total score = 8)**						
Post-test	89.3%	7.14	1.03	(3.69–3.95)	37.350	< 0.001
Pre-test	47.8%	3.82	1.32	(7.04–7.24)		
Differences	41.5%	3.32	1.78	–		
**Knowledge about screening of breast cancer (total score = 5)**						
Post-test	79.6%	3.98	0.71	(1.76–1.87)	47.241	< 0.001
Pre-test	36.4%	1.82	0.55	(3.91–4.05)		
Differences	43.2%	2.16	0.92	–		
**Knowledge about process of breast self-examination (total score = 5)**						
Post-test	78.8%	3.94	0.93	(1.39–1.76)	23.653	< 0.001
Pre-test	31.4%	1.57	1.86	(3.85–4.03)		
Differences	47.4%	2.37	2.00	–		

*SD* Standarddeviation

^a^Paired t-test

**Table 3:** Assessment of percentage of changes in practice of breast self-examination (pre-test vs post-test)

Variables	Pre-test		Post-test		Percentage of correct changes	Mc-Nemartest* ***p***-value
	n	% (95% CI)	n	% (95% CI)		
**Breast self-examination practice**						
Yes	85	21.3 (17.5–25.5%)	135	33.8 (29.2–38.5%)	12.5%	< 0.001
No	315	78.8 (74.5–82.5%)	265	66.3 (61.5–70.8%)		

## Discussion

The present study found a significant change of knowledge of breast cancer and BSE practices following an educational intervention among undergraduate female students in Bangladesh. Fifteen days after our educational intervention, all participants were re-contacted for a post-test survey. This 100% response rate for the post-test survey is understandable because all the respondents of this study were residential students of the university who were residing at their own dormitories during the full period of study. Still, some participants were not present for the post-test session. To trace them back we had to contact them via their contact numbers personally which was obtained from them with their full informed consent by assuring the confidentiality and thus got the post-test data from all of the participants.

After the educational intervention and 15 days interval, we assessed the knowledge level of the same respondents. Correct answers were delivered by the majority of the respondents about each question in the post-test session. This is consistent with several studies conducted in Egypt, Iran, İzmir (a city of Turkey), and Sivas (a city in the Central Anatolia of Turkey) [18–21]. In these studies, the knowledge level on breast cancer symptoms, risk factors, treatment, prevention, screening methods and practice of BSE were significantly increased after educational session among the respondents. Yilmaz et al. have showed that the mean knowledge score for correct risk factors and correct screening methods were increased from 3.65 ± 2.86 to 9.37 ± 3.10 (total score 12) and 5.45 ± 1.98 to 8.10 ± 1.19 (total score 6), respectively from pre-test to post-test and it supports out finding [21]. Aziz et al. and Rezaein et al. have also revealed in their studies about significant increase in correct knowledge changes about symptoms, risk factors, prevention, and early detection of screening methods after a successful educational/training intervention [18, 19]. The changes in the knowledge level were significant in a previous study by Ceber et al. and correct percentage of changes was higher in the post-test [20]. These findings confirm the significant improvement in knowledge about breast cancer in our study.

In the present study, the knowledge of BSE also increased significantly. The mean difference in the knowledge about process of BSE (total score = 5) was 2.37 ± 2.00 (*p* < 0.001). This finding is consistent with a previous study by Ceber et al. where the knowledge level on BSE was higher in the experimental group who received an educational session (control 6.13 ± 0.91 vs. experimental 7.03 ± 0.84) [20]. The study by Aziz et al. with Egyptian women aligns with our finding e of a significant improvement in the knowledge of BSE after the educational intervention (the mean difference from pre-test to post-test was 0.71 ± 0.46) [18]. Another study with female students on the effect of BSE education in Turkey by Beydağ et al. found a similar result, whereby the knowledge on BSE was significantly increased (the knowledge score was 43.2 ± 10.6 before and 68.4 ± 10.5 after the BSE education (*p* < 0.05)) [22]. These studies support our finding on the increased knowledge of BSE post-intervention. With regard to the practice of BSE, our study found significant changes from 21.3% (pre) to 33.8% (post-intervention) (*p* < 0.001) (Table 3). Similarly, a number of other studies have also found similar results. In a study in Yazd University, Iran, it was found that before training, 62.86% of the women did not perform BSE but, after training this percentage decreased to 33.57% (*p* < 0.001) [23]. Similarly, Ozturk et al. found that the percentage of participants who regularly performed BSE in the intervention group increased from 19.0 to 61.3%, (this increase was statistically significant) while the control group participants remained stable at 39.7% (the difference between intervention and control groups was statistically significant) [24]. In a different study, after an educational intervention, there was an increase from 70 to 75%70% of women practicing BSE (*t* = 9.84, *p* < 0.001) [18].

All these studies discussed above that showed that educational interventions can lead to positive changes in knowledge, awareness and practice towards breast cancer and BSE. However, the time interval between the educational intervention and post-test survey, as well as the number of educational sessions, can impact the outcome. The difference in knowledge and practice were statistically higher in our study after educational intervention but the changes in percentage were not satisfactory (ranging from 12.5 to 47.4%.) if we compare with some other studies conducted on women worldwide. Studies in Iran, Egypt and Pakistan have showed higher percentage of changes in knowledge and practice than our study [9, 19, 20]. One possible reason of that could be greater time interval than our study and follow-up sessions. More time-interval between the intervention and post-test could increase the percentage of practice of BSE. Also, inclusion of follow-up of the sessions during the interval phase could increase the percentage of positive changes in knowledge and practice. These factors should be taken into consideration while designing similar studies among different population groups.

Given the fact that this study was conducted with university students (1st year undergraduates to post-graduates) in combination with an efficient, flexible and attractive educational session, this is justifiable that they understood the information provided at the educational session on breast cancer and practice of BSE easily, indicate the successful outcome of the educational session that was conducted in this study. Though all changes in the knowledge and practice of breast cancer and BSE were statistically significant, the percentages of changes were not satisfactory at all. It was expected to changes more than 50%. However, our findings demonstrated the changes ranging from 12.5 to 47.4%.

Even though our study was with university students, all women regardless of their socio-economic or demographic conditions need to be educated about breast cancer and breast cancer screening methods. This education should be culturally appropriate and targeted towards individual population so that it can create greater impact.

### Limitations

The study had only 15 days interval between the pre-test, educational session and the post-test assessment, increasing the chance of recall bias. If more time interval could be given, that might have impacted the outcomes. No follow-up session of the educational intervention on breast cancer and BSE could be given, also due to time constraints. Respondents were given no reminder to practice BSE during the interval phase. All the data were self-reported by the respondents and no verification could be done to assess the accuracy of the data given by respondents whether the claim of practicing BSE were true or not. The quality of BSE practice could not be assessed. So, it is unknown that if the respondents who are claiming to actually practicing BSE were being able to do it properly or not. Moreover, we also cannot claim any generalizability to other groups of women in Bangladesh given that our sample are highly educated.

## Conclusion and recommendation

The findings indicated that women’s knowledge regarding breast cancer warning symptoms, risk factors, treatment, prevention, effective screening methods and practice of BSE were sub-optimal at baseline. The results of the post-test of this study suggest that women’s knowledge was significantly increased after providing an educational intervention. However, educational sessions should be continued because increased knowledge level is important to change behavior about early diagnosis for breast cancer. This study concludes that the educational program on breast cancer and BSE has been effective in improvement of knowledge and BSE practice levels of women. A future study with larger and diversified population is recommended to assess the effectiveness in different population groups of women and monitor the changes in awareness and practice of breast cancer and breast cancer screening.

## Supplementary Information

**Additional file 1: Table S1.** Knowledge about symptoms of breast cancer. **Table S2.** Knowledge about risks of breast cancer. **Table S3.** Knowledge about treatment of breast cancer. **Table S4.** Knowledge about prevention of breast cancer. **Table S5.** Knowledge about screening of breast cancer. **Table S6.** Knowledge about process of breast cancer.

## Data Availability

The data described in this article can be freely and openly accessed at Mendeley Data: 10.17632/jdvyg74sbv.1.

## Abbreviation

BSE: Breast self-examination
